# Building Digital Trust in East Africa: The Role of National ID and ICT Policy in Somalia’s Digital Transformation

**DOI:** 10.12688/f1000research.182539.2

**Published:** 2026-09-10

**Authors:** Mohamed Adam Isak, Abdulkadir Jeilani Mohamud

**Affiliations:** 1 Department of Computer Science, Somali National University, Somali National AI Center, Mogadishu, Banaadir, Somalia; 2 Department of Computer Science, Somali National University, Mogadishu, Somalia

**Keywords:** Digital Identity, ICT Policy, Cybersecurity, Somalia’s Digital Transformation, E-government.

## Abstract

Trusted digital identities sit at the heart of any modern, inclusive financial system. This paper investigates Somalia’s transition from a fragmented digital landscape to a secure, integrated ecosystem through the lens of a “Three Pillars” framework: Robust Digital Identity, Comprehensive ICT Policy, and Proactive Cybersecurity Safeguards. Utilizing a qualitative multi-method approach, the study triangulates fifteen semi-structured interviews with regulators, financial experts, and civil society against a rigorous analysis of foundational policies, including the Data Protection Act (2023) and the Cybersecurity Law (2026).

Results indicate that Somalia’s digital identity ecosystem—comprising e-Aqoonsi, HUBIYE, and the Certificate Delivery System (CDS)—serves as a technical anchor for financial inclusion, significantly bolstered by a July 2025 Central Bank mandate requiring National IDs for all banking transactions. These tools are supported by legal scaffolding under the Data Protection Authority and proactive security guardrails provided by the Somalia Computer Incident Response Team (SOMCIRT).

However, comparative analysis reveals that while Somalia has achieved technical parity with East African Community (EAC) peers in biometric capabilities, it remains an outlier due to its reliance on foreign data hosting, which poses risks to national data sovereignty. To realize Somalia’s Centennial Vision 2060, the study concludes that the nation must bridge implementation gaps in rural digital literacy and technical capacity while transitioning toward local data infrastructure. The paper proposes a strategic roadmap for regional harmonization and enhanced institutional coordination to sustain public trust in the emerging digital economy.

## Introduction

Across East Africa, the rapid growth of digital financial services is transforming how people save, transact, and access credit—especially among populations previously excluded from formal financial systems ( World Bank, 2025). Mobile money platforms, agent banking, and digital wallets have become powerful tools for economic empowerment ( East African Community Secretariat, 2021). Yet, behind this progress lies a critical concern: the issue of digital trust ( OECD, 2020). Many East African countries still struggle with fragmented identity verification systems, inconsistent cybersecurity protections, and mismatched regulatory frameworks ( World Bank, 2023). In Somalia, the situation is even more complex. Prolonged instability and limited infrastructure have slowed the development of a secure, inclusive digital identity ecosystem ( Centre for Internet and Society, 2023), although the Data Protection Act (2023) and the newly established Data Protection Authority now provide a legal framework to enhance trust and privacy protections ( Federal Republic of Somalia, 2017, 2019).

Recognizing this, both national and regional actors are working to reverse course. The East African Community (EAC), for instance, is pushing for broader digital integration through strategic initiatives like the World Bank-supported Eastern Africa Regional Digital Integration Project (EARDIP) ( World Bank, 2025). These efforts highlight a critical understanding: for digital systems to succeed, they must not only be technologically sound, but also rooted in local realities, human rights principles, and user trust.

In Somalia, tangible progress is underway through the National ICT Policy & Strategy 2019–2024 ( Federal Republic of Somalia, 2019), the National Communications Law 2017 ( Federal Republic of Somalia, 2017), and the ICT Regulatory Transformational Strategy 2023–2027 ( National Communications Authority, 2025). These documents collectively aim to expand affordable internet access, promote e-Government services, reinforce regulatory frameworks, introduce enforceable data protection measures under Law No. 005/2023, and establish secure digital ID systems. Prominent initiatives such as e-Aqoonsi, HUBIYE, and the Certificate Delivery System (CDS) illustrate this commitment ( NIRA, 2024a, 2024b, 2024c).

Importantly, these digital reforms align with Somalia’s Centennial Vision 2060, the nation’s long-term strategy to become a stable, inclusive, and middle-income country by the time it reaches its 100th year of independence. Vision 2060 places digital transformation and institutional trust at the heart of economic development, emphasizing the role of ICT in service delivery, governance, and citizen empowerment. However, achieving these ambitions depends on addressing foundational issues—such as fragmented identity systems, weak cybersecurity infrastructure, and low public trust—which this paper seeks to explore.

This paper proposes that the construction of digital trust in Somalia is built upon three essential pillars: Robust Digital Identity, Comprehensive ICT Policy, and Proactive Cybersecurity Safeguards.

▪ Pillar 1: Digital Identity serves as the foundation, utilizing systems like e-Aqoonsi, HUBIYE, and the Certificate Delivery System (CDS) to authenticate users and reduce fraud.
▪ Pillar 2: ICT Policy provides the legal scaffolding, primarily through the Data Protection Act (2023), which ensures the lawful processing of personal information.
▪ Pillar 3: Cybersecurity acts as the security guardrail, operationalized by the Cybersecurity Law (2026) and the Somalia Computer Incident Response Team (SOMCIRT) to protect critical infrastructure.

This paper examines how these three pillars, when integrated and aligned with regional efforts, can contribute to building digital trust and enabling financial inclusion—not only in Somalia but throughout the East African region.

## I. Literature Review

### A. Theoretical Foundations of Digital Transformation

Before turning to sector-specific literature, it is useful to situate Somalia’s experience within broader theoretical debates on digital transformation (DT). Vial (2019) conceptualizes DT as a process triggered by digital technologies that provokes significant changes to an organization’s or a society’s value-creation paths through evolving structures, processes, and stakeholder relationships — a framing that captures why Somalia’s shift from fragmented, paper-based identity verification toward an integrated digital ecosystem (e-Aqoonsi, HUBIYE, CDS) constitutes DT in the fullest sense, rather than a narrow technology upgrade. In the public sector specifically, Mergel, Edelmann, and Haug (2019) show that DT is best understood as a cultural, organizational, and technological change grounded in strategy, echoing this study’s finding that Somalia’s three pillars must be institutionally — not merely technically — aligned to build trust.

The socio-technical and institutional dimensions of this transition are further illuminated by Fountain’s (2001) technology enactment framework, which argues that the developmental outcomes of ICT are not determined by the technology itself but are “enacted” through the institutional arrangements, organizational routines, and bureaucratic capacities of the adopting state — a lens that helps explain why Somalia’s technical parity with EAC peers in biometric capability has not yet translated into equivalent institutional coordination or rural uptake. Complementing this, Meijer and Bolívar (2016) emphasize that digital governance is inherently a multi-actor, network-based process requiring collaboration among public agencies, private telecom and financial operators, and civil society — consistent with this study’s finding that Somalia’s digital identity ecosystem depends on coordination across NIRA, the Central Bank, private banks, and telecom-based mobile money operators. Finally, Heeks (2020) situates such reforms within the broader “digital-for-development” (ICT4D 3.0) paradigm, which stresses that digital public infrastructure in fragile and low-resource states carries distinct risks around data governance, platform dependency, and datafication — directly relevant to this paper’s concerns over foreign data hosting and national data sovereignty.

These theoretical perspectives also extend into the financial-sector literature relevant to Somalia’s Sharia-compliant banking system. Alkhwaldi (2025) identifies digital trust, financial literacy, and perceived quality-of-life benefits as key antecedents of fintech adoption, reinforcing this study’s central claim that trust — not merely technical availability — determines whether digital identity tools translate into financial inclusion. Abu-AlSondos et al. (2024) survey the opportunities and organizational challenges of DT across business sectors, including talent shortages, legacy-system constraints, and change-management barriers that parallel the institutional-capacity and coordination gaps identified among Somali regulators and financial institutions in this study. Most directly, Shehadeh et al. (2023) provide empirical evidence that DT improves operational efficiency and competitive advantage specifically within Islamic banks — a finding with direct bearing on Somalia’s predominantly Sharia-compliant financial sector and its dependence on trusted digital identity for KYC and account-opening processes. Together, these studies extend the paper’s Three Pillars framework beyond its policy and legal focus, grounding it in wider theoretical debates on socio-technical change, institutional capability, and digital government evolution.

### B. Financial Inclusion and Digital Payments in the EAC

Mobile money platforms, notably Kenya’s M-PESA, have significantly broadened financial access for the unbanked ( Vodafone Group, 2025). In Somalia, local mobile payment services such as EVC Plus by Hormuud Telecom and E-Dahab by Dahabshiil also play a crucial role in daily financial transactions. These platforms are widely adopted across the country, enabling users to send and receive money, pay bills, top up airtime, and access small-scale digital finance. Their accessibility—even in remote areas—has made them essential tools for driving financial inclusion in fragile settings.

The transformative impact of mobile money on financial inclusion has been extensively documented in Kenya. Jack and Suri (2014) found that M-PESA reduced transaction costs, improved risk-sharing among households, and enhanced financial resilience, particularly among low-income populations. These findings demonstrate the broader potential of mobile money platforms to promote inclusive economic participation and provide valuable lessons for other East African countries, including Somalia.

However, despite this progress, regional interoperability and regulatory inconsistencies continue to hinder cross-border financial integration ( East African Community Secretariat, 2024). The Eastern Africa Regional Digital Integration Project (EARDIP) aims to address these barriers by harmonizing payment systems and e-commerce regulations across the region ( World Bank, 2025).

In Somalia, digital finance is underpinned by the National ICT Policy & Strategy 2019–2024, which prioritizes affordable ICT access and digital literacy as prerequisites for financial inclusion ( Federal Republic of Somalia, 2019). Complementary reports, including the ICT Sector Investment Study ( Somalia Investment Promotion Office, 2022) and the World Bank Digital Economy Report ( World Bank, 2022), stress the importance of regulatory reform, public-private partnerships, and digital infrastructure investment to unlock inclusive financial services.

Launched in 2007, M-PESA remains the region’s most successful fintech innovation. With more than 60 million users across eight countries—including Kenya, Tanzania, Ghana, and Ethiopia—M-PESA enables mobile-based transactions such as money transfers, bill payments, and credit access through a network of over 660,000 agents ( Vodafone Group, 2025). Its success offers insights for replication and scaling across the Horn of Africa, particularly in fragile markets like Somalia.

### C. National ID Systems as a Tool for Trust

Robust national ID systems are critical for secure identity verification and trusted access to public and private services ( World Bank, 2019a, 2019b). Countries such as Rwanda and Uganda have implemented successful digital ID frameworks through platforms like IREMBO and NIRA, which enable biometric-based authentication for a range of services ( Republic of Rwanda, 2015; Republic of Uganda, 2015).

Somalia’s National Identification Number (NIN) initiative seeks to emulate and localize these models by linking digital IDs with financial services. The National ICT Policy & Strategy 2019–2024 underscores digital identity as central to e-Government and financial inclusion ( Federal Republic of Somalia, 2019), while the National Communications Law 2017 ( Federal Republic of Somalia, 2017) and the Somalia Data Protection Act 2023 provide a legal basis for consumer protection, data privacy, and lawful processing of personal information in ID-linked services.

A major regulatory shift occurred in July 2025, when the Central Bank of Somalia (CBS), in partnership with the National Identification and Registration Authority (NIRA), mandated that all individuals must present a government-issued National ID to open or renew bank accounts, effective 1 September 2025. This directive establishes a standardized identity verification framework across all financial institutions and closes long-standing gaps in digital trust and user authentication ( Somali National News Agency [SONNA], 2025a).

### D. Cybersecurity in Digital Finance

As digital financial ecosystems expand, exposure to cyber threats—including fraud, data breaches, and system disruptions—also increases ( OECD, 2020). Weak cybersecurity frameworks can discourage user trust and limit the adoption of digital financial services. Regional initiatives such as the Eastern Africa Regional Digital Integration Project (EARDIP) emphasize the importance of strengthening cybersecurity and data governance across the EAC ( Federal Republic of Somalia, 2017).

In Somalia, earlier assessments identified major gaps in cyber readiness, prompting reforms and incident response planning ( Federal Government of Somalia, 2020). This has advanced with the Cybersecurity Law (2026), which establishes a national framework for protecting infrastructure, securing systems, and managing cyber incidents ( National Communications Authority [NCA], 2026a). The law also defines institutional roles and response mechanisms.

Complementing this, Somalia has operationalized the Somalia Computer Incident Response Team (SOMCIRT) as the national center for detecting and responding to cyber threats ( National Communications Authority [NCA], 2026b), marking a shift toward a more coordinated approach.

However, challenges remain, including limited technical capacity, shortage of skilled professionals, and weak institutional coordination. Addressing these gaps is essential to ensure secure digital financial systems and sustain public trust.

### E. Gaps in Literature

Although digital ID systems and financial technologies have been widely studied, few academic works focus on the intersection of ID systems and cybersecurity in fragile contexts such as Somalia. Regional strategies like the EAC E-Commerce Framework and Somalia’s e-Government Strategy Terms of Reference call for deeper research into public service digitization and citizen participation—areas still underrepresented in scholarly literature ( OECD, 2020).

## II. Conceptual Framework

The conceptual framework for this study posits that digital trust is not a standalone technological achievement but a multi-dimensional construct emerging from the convergence of three foundational pillars: Robust Digital Identity, Comprehensive ICT Policy, and Proactive Cybersecurity Safeguards. In the context of a fragile state like Somalia, these pillars provide the necessary technical, legal, and operational infrastructure required to move from a cash-based, informal economy to an inclusive digital financial ecosystem.

### Pillar 1: Robust Digital Identity (The Foundation)

At the base of the framework is Digital Identity, which serves as the primary mechanism for establishing “who is who” in the digital realm. In Somalia, this is operationalized through the National Identification and Registration Authority (NIRA) and its ecosystem of platforms:

▪ e-Aqoonsi: Provides biometric-based authentication (facial recognition and fingerprints) to ensure unique identity and reduce fraud. The platform allows citizens to access services remotely, including bank accounts, SIM registration, and welfare programs ( SONNA, 2025b; NIRA, 2024a).
▪ HUBIYE: Acts as a real-time verification gateway, allowing financial institutions and government agencies to validate identities against the national database. The platform supports user-consented data sharing and is designed for interoperability, fostering trust in cross-sectoral digital transactions ( SONNA, 2025b; NIRA, 2024a).
▪ Certificate Delivery System (CDS): Ensures the authenticity of foundational documents, such as birth certificates, which are necessary for tiered Know-Your-Customer (KYC) processes. It incorporates features like QR codes and digital signatures to ensure document authenticity and reduce forgery risks, improving public sector transparency and service delivery ( MoIFAR, 2023). The framework views these tools as the “anchor” for trust, particularly following the 2025 Central Bank mandate requiring a National ID for all banking transactions.

### Pillar 2: Comprehensive ICT Policy (The Legal Scaffolding)

The second pillar provides the legal scaffolding necessary to protect user rights and regulate the behavior of digital actors. Without enforceable laws, technological tools like digital IDs can lead to privacy concerns or data misuse. This study focuses on:

▪ The Data Protection Act (2023): This law establishes the legal basis for the lawful processing of personal information and creates the Data Protection Authority to oversee compliance.
▪ National ICT Policy & Strategy 2019–2024: This framework prioritizes affordable access and digital literacy as prerequisites for trust.
▪ ICT Regulatory Transformational Strategy 2023–2027: This aligns domestic goals with regional standards, ensuring that Somalia’s digital growth remains compatible with the broader East African Community (EAC).

### Pillar 3: Proactive Cybersecurity Safeguards (The Security Guardrail)

The third pillar acts as the security guardrail, protecting the system from external threats and system disruptions that could otherwise erode public confidence. The framework identifies two critical components recently introduced in Somalia:

▪ Cybersecurity Law (2026): This provides the legal mandate for protecting critical national infrastructure and defines the institutional roles for managing cyber incidents.
▪ Somalia Computer Incident Response Team (SOMCIRT): This serves as the operational center for detecting, preventing, and responding to threats such as phishing and data breaches. By transitioning from a reactive to a proactive security stance, Somalia aims to mitigate the risks associated with foreign-hosted data systems and enhance national data sovereignty.

Digital trust emerges through the systematic integration of three pillars as shown in Figure 1: Digital Identity as a technical anchor, ICT Policy as legal scaffolding for data rights, and Cybersecurity as a protective guardrail. This convergence facilitates Somalia’s transition to an inclusive digital financial ecosystem. Harmonizing these reforms with EAC standards and Vision 2060 enables expanded access to Sharia-compliant finance and remittances for marginalized groups. Thus, digital transformation establishes a trusted foundation for economic empowerment and national data sovereignty.

**Figure 1.**
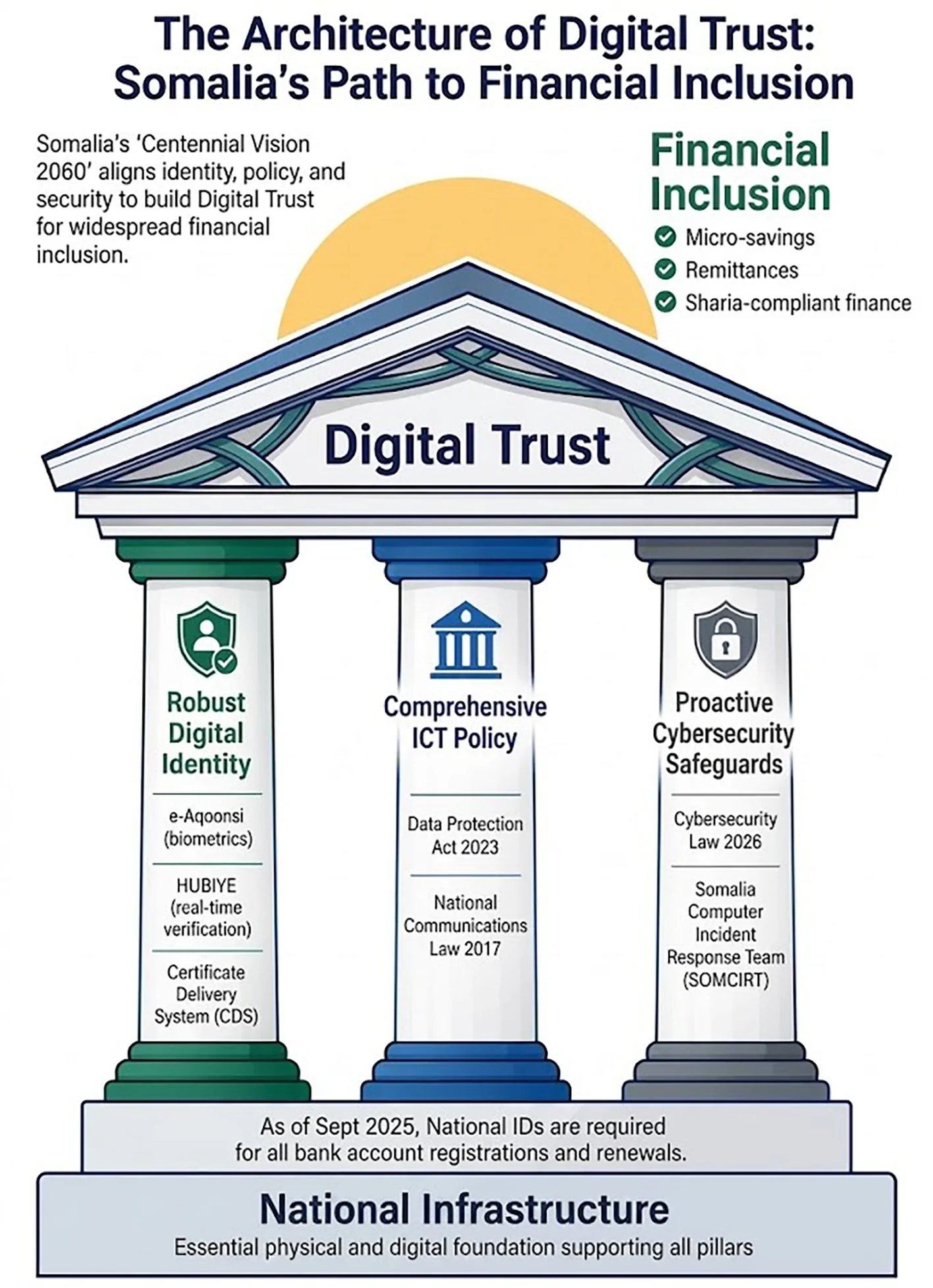
Temple of Trust model. *Note*: Conceptual framework developed by the author; visualization generated with assistance from Google Gemini ( Google, 2025).

## III. Data and Methodology

This study utilizes a qualitative, multi-method approach to capture the technical and social complexities of digital trust in Somalia. By combining primary insights from key stakeholders with a rigorous analysis of policy documents, the research provides a comprehensive view of how identity systems and regulations intersect to foster financial inclusion.

### A. Research Design

The researchers followed a qualitative, multi-method approach, combining semi-structured interviews with document analysis, to capture firsthand insights and policy trends shaping digital trust in Somalia, within the broader East African context.

### B. Data collection

• **Semi-Structured Interviews**

To capture nuanced perspectives on digital trust, fifteen key informants—including consultants, IT engineers, digital finance experts, bank staff, and civil society representatives—were interviewed between June 2025 and Jan 2026, via in-person and virtual formats using a conversational style with open-ended questions. To ensure the integrity and candor of the data, all participants provided informed consent regarding anonymity and confidentiality, with interviews held in neutral, private settings. Researchers’ bias was mitigated through the systematic documentation of reflections in detailed field notes.

• **Policy review**

Primary interview data was triangulated with a rigorous review of foundational Somali legal and strategic frameworks. This included the National ICT Policy & Strategy 2019–2024, National Communications Law 2017, and ICT Regulatory Transformational Strategy 2023–2027. Critically, the review also examined the Data Protection Act (2023) and the Cybersecurity Law (2026) to evaluate the legal scaffolding of the digital ecosystem. These documents were analyzed alongside comparative features of other East African Community (EAC) national ID frameworks, as summarized in Table 1.

**Table 1.** Comparative Analysis of National Digital Identity Systems in Somalia and Selected East African Countries.

Country	Introduced	Biometrics	Smart Card	Mobile ID	Data Protection Law	Cybersecurity Law	Financial Integration	Data Hosting	Cross-Border Interoperability
Somalia	2022 (Pilot)	Yes	Yes	Yes	Yes	Yes	Full	Foreign	Planned
Kenya	2013	Yes	Yes	Yes	Yes	Yes	Full	Local	Yes
Uganda	2014	Yes	Yes	No	Yes	Yes	Full	Local	Yes
Rwanda	2015	Yes	Yes	Yes	Yes	Yes	Full	Local	Yes
Tanzania	2013	Yes	Yes	No	Yes	Yes	Full	Local	Yes
Burundi	2016	Yes	No	No	No	No	Partial	Local	Planned
South Sudan	2017 (Pilot)	Yes	No	No	No	No	Partial	Foreign	Planned
DRC	2018 (Pilot)	Yes	No	No	Draft	No	Partial	Local	Planned

*Notes:* Biometrics = fingerprint, facial recognition, or iris data used in identity verification.

Mobile ID = availability of digital identity services through mobile applications or mobile-based credentials.

Financial Integration = linkage with banking services, SIM card registration, digital payments, and government services.

Local Hosting = data stored within national data centers; Foreign Hosting = data stored outside the country.

Cross-Border Interoperability refers to the ability of national digital ID systems to integrate with regional identity frameworks such as the East African Community (EAC), COMESA, or ECCAS initiatives.

*Source:* Compiled by the authors from official government digital identity programs, national ICT policies, and regional digital governance reports (2025).

### C. Ethical Considerations

This study was conducted in accordance with established ethical principles for research involving human participants, including voluntary participation, confidentiality, participant autonomy, and responsible data management. The research was designed as a minimal-risk qualitative study based on expert interviews addressing digital trust, cybersecurity, and financial inclusion within the Somali context.

In accordance with the institutional research guidelines applicable to the authors’ affiliated institution, formal ethical approval was not required for this study, as it involved non-sensitive expert perspectives, did not include vulnerable populations, and posed no foreseeable risk to participants. Nonetheless, the study was conducted with strict attention to ethical responsibility, ensuring the protection, dignity, and rights of all participants throughout the research process.

Prior to participation, all respondents were fully informed about the objectives, scope, and academic purpose of the study, as well as the voluntary nature of their involvement. Participants were also informed that interviews would be audio-recorded solely for research and transcription purposes.

Informed verbal consent was obtained from all participants before each interview. Verbal consent was selected over written consent due to the specific nature of this research, which involved discussions of institutional, policy, and governance-related issues in Somalia. Many participants held professional positions in government or affiliated institutions, and requesting signed consent documents could create hesitation or perceived professional risk when expressing their views. Such formality could also affect the openness and depth of responses, which were critical for capturing authentic expert perspectives. Given that the study did not involve personal or sensitive individual data and relied on high-level professional insights, verbal consent was considered the most appropriate approach to ensure both ethical compliance and participant trust.

To ensure transparency and accountability, verbal consent was explicitly confirmed and documented at the beginning of each interview through audio recording and researchers’ field notes. Participants provided consent for participation, audio recording, and the use of anonymized data for academic research and publication.

Interviews were conducted in secure and neutral settings, and no personally identifiable information was included in the analysis or reporting of findings. Audio recordings, transcripts, and field notes were securely stored and accessed only by the researchers for academic purposes. Participants were informed of their rights, including the right to decline participation, refuse to answer any question, or withdraw from the study at any time without consequence.

### D. Data Analysis

Audio-recorded interviews were transcribed and systematically analyzed using NVivo software through a specialized two-phase coding process. First, thematic coding was applied to extract nuanced stakeholder perspectives on the “Three Pillars” of digital trust: identity authentication (e-Aqoonsi/HUBIYE), proactive cybersecurity (SOMCIRT), and public trust in Sharia-compliant financial systems. Concurrently, foundational policy documents—including the Data Protection Act (2023) and the Cybersecurity Law (2026)—underwent structural coding to map regulatory objectives against identified implementation gaps, specifically regarding data sovereignty and rural digital literacy. Finally, the synthesis of primary interview data and secondary policy analysis was triangulated with regional comparisons from the East African Community (EAC) to yield evidence-based, context-sensitive recommendations aligned with Somalia’s Centennial Vision 2060.

## IV. Results and Discussion

Synthesizing results from stakeholder interviews and comprehensive analysis of policy documents, this section evaluates Somalia’s digital transition through the established three-pillar framework.

### Pillar 1: Digital Identity as a Technical Anchor

National ID systems are widely regarded as essential infrastructure for secure digital transactions across the East African Community (EAC) ( Gelb & Diofasi Metz, 2018; UNECA, 2019). In Somalia, the launch of e-Aqoonsi marked a significant turning point in accessibility; as noted by a Somali IT engineer in 2025, “The e-Aqoonsi app has enabled remote communities to obtain digital IDs without traveling to cities”. While biometric authentication and real-time verification through HUBIYE have enhanced institutional trust, stakeholder interviews indicated that public awareness of these capabilities remains notably low in rural areas.

A critical regulatory milestone occurred in July 2025, when the Central Bank of Somalia mandated the use of a government-issued National ID for all banking transactions, effective September 2025,. This directive standardized identity verification across the financial sector and addressed long-standing authentication gaps. Finally, the Certificate Delivery System (CDS) streamlines the issuance of official documents, utilizing digital signatures and QR codes to reduce opportunities for corruption and exclusion ( Gelb & Diofasi Metz, 2018; UNECA, 2019), ( NIRA, 2024a). Together, these platforms establish the technical “anchor” necessary for expanding financial inclusion and building a resilient digital economy.

While comprehensive, disaggregated enrollment data were not made available to the researchers directly by NIRA, independent secondary reporting offers a useful proxy for the pace of adoption. As of November 2025, NIRA’s Director-General reported that enrollment in the national ID programme had surpassed one million citizens, with more than two dozen public- and private-sector services — including banking and telecommunications — integrated for real-time authentication ( Biometric Update, 2025). By January 2026, this figure represented approximately 7% of the government’s stated target of 15 million registrations by 2029, with only around one-fifth of the estimated US$125 million implementation budget secured to date ( Biometric Update, 2026). These figures should be read as indicative rather than precise, being drawn from public statements rather than NIRA’s own disaggregated statistics; nonetheless, they corroborate this study’s qualitative finding that rural and under-resourced populations remain substantially under-registered, and that sustained donor and government investment will be required to close this gap before Vision 2060.

### Pillar 2: ICT Policy as Legal Scaffolding

The research highlights a critical shift from policy drafting to active legal enforcement, providing the “scaffolding” necessary to protect user rights.

### The Data Protection Framework

The enactment of the Data Protection Act (Law No. 005/2023) and the establishment of a functional Data Protection Authority have created a legal basis for the lawful processing of personal information. This aligns Somalia with regional standards and addresses long-standing privacy concerns by introducing enforceable measures. Emphasizing the practical application of these protections, a NIRA staff member noted: “Consent features in the e-Aqoonsi app help build trust, but ongoing education is still needed”.

### Alignment with Vision 2060

These reforms are central to Somalia’s Centennial Vision 2060, which prioritizes digital transformation as a means of governance and citizen empowerment. By placing institutional trust at the heart of economic development, the policy framework ensures that technology serves the broader goal of national stability. A Somali regulator underscored the importance of this human-centric approach, stating: “The success of e-Aqoonsi hinges not just on technology **,** but on whether citizens believe their data is safe and that the system works in their favor”.

### Regional Interoperability

As shown in Table 1, while Somalia has historically lagged behind East African Community (EAC) peers like Kenya and Rwanda, its recent legislative surge has significantly narrowed the regulatory gap,. However, challenges remain as cross-border interoperability and recognition of digital credentials currently remain in the “planned” phase rather than being fully active.

### Pillar 3: Proactive Cybersecurity as a Guardrail

The transition from a “reactive” to a “proactive” security stance is marked by the introduction of institutional safeguards designed to protect critical infrastructure. As a cybersecurity expert reported during the research: “Without a proactive cybersecurity policy, we’re always reacting to threats like phishing and SIM swaps”.

Legislative and Operational Launch: The Cybersecurity Law (2026) provides the legal mandate for securing information systems, while the Somalia Computer Incident Response Team (SOMCIRT) serves as the operational center for detecting and responding to national cyber threats. This shift allows Somalia to complement regional efforts within the East African Community (EAC), where countries have made significant strides in establishing CERTs and consumer protections.

The Data Sovereignty Concern: A recurring theme in stakeholder interviews is the risk associated with foreign-hosted data systems, which leaves the digital ecosystem vulnerable to external data misuse. An advisor at the Ministry of Communications and Technology emphasized that: “Ongoing efforts toward data localization, including the development of local data hosting infrastructure, are expected to strengthen national cybersecurity by reducing reliance on foreign-hosted systems and improving control over sensitive information”. Experts emphasized that until Somalia develops local data centers, its national sovereignty remains directly impacted by this technical reliance.

The findings suggest that digital trust in Somalia is not merely a byproduct of technology but is built through the alignment of these three pillars. As summarized in Table 1, Somalia has successfully matched its EAC peers in biometric and mobile ID capabilities. However, the study concludes that the final step toward a resilient digital economy requires moving from foreign hosting to local data sovereignty and ensuring that rural citizens believe their data is safe through ongoing education. As a Somali regulator emphasized: “The success of e-Aqoonsi hinges not just on technology, but on whether citizens believe their data is safe and that the system works in their favor”. This trust is further reinforced by transparent “consent features” within platforms like e-Aqoonsi, which NIRA staff noted “help build trust, but ongoing education is still needed”.

### Comparing EAC National ID Systems

A comparative review indicates that Somalia has historically trailed its East African peers in data protection, cybersecurity regulations, and cross-border interoperability (see Table 1). While nations such as Kenya ( NIRA, 2024b), Uganda ( Republic of Rwanda, 2015), ( Republic of Uganda, 2015), Rwanda ( Republic of Rwanda, 2015), the Democratic Republic of Congo (DRC) ( World Bank, 2019a), ( World Bank, 2019b), and Tanzania ( Somalia Investment Promotion Office, 2022), ( Gelb & Clark, 2013) are actively piloting interoperable digital ID systems, Somalia’s regulatory environment remained in a developmental drafting phase for a prolonged period.

However, the enactment of the Data Protection Act (Law No. 005/2023), the establishment of a functional Data Protection Authority, and the approval of the Cybersecurity Law (2026) have significantly reinforced the legal scaffolding and security guardrails necessary for institutional trust ( NIRA, 2024a, 2024b, 2024c; SONNA, 2025a). This legislative progress establishes a robust foundation for personal data management, though Somalia remains the only regional actor identified as utilizing foreign data hosting rather than local infrastructure.

### Implementation Gaps and Findings

Despite the technical and legislative milestones achieved through the three-pillar framework, research findings identify six critical implementation gaps that hinder the full realization of digital trust in Somalia.

▪ Infrastructure Inadequacy: Rural regions continue to face significant deficits in digital infrastructure, which restricts access to online identity services and limits the equitable distribution of financial tools.
▪ Digital Literacy Deficits: Awareness regarding digital rights and the functionality of platforms like HUBIYE remains low, particularly among marginalized populations. This lack of “digital citizenship” creates a barrier to effective system utilization.
▪ Biometric Enrollment Barriers: Technical challenges during the enrollment phase—ranging from hardware malfunctions to environmental factors—frequently affect the coverage, accuracy, and overall efficiency of the national ID database.
▪ Regulatory Enforcement Hurdles: While the Data Protection Act (2023) and Cybersecurity Law (2026) are now in force, an implementation gap persists. Institutional coordination between the Data Protection Authority and SOMCIRT is still evolving and requires sustained investment in human capacity.
▪ Data Sovereignty Risks: A recurring concern among stakeholders is the ongoing reliance on foreign-hosted data systems. This technical dependency raises significant national security concerns and remains a primary hurdle to achieving regional digital sovereignty.
▪ Data Transparency Gaps: Although e-Aqoonsi registrations are increasing, comprehensive empirical data regarding its direct impact on fraud reduction and financial inclusion remains unavailable.

The study concludes that while Somalia shows significant promise in digital identity transformation, its overall cybersecurity readiness and legal enforcement mechanisms still lag behind its EAC neighbors. As a Somali regulator underscored, the ultimate success of these initiatives hinges not just on technology, but on whether citizens believe their data is safe. Addressing these systemic gaps is essential to move from “digital potential” to a resilient, integrated, and inclusive digital economy.

## V. Conclusion and Policy Implications

The findings of this study demonstrate that integrating digital identity systems with robust ICT and cybersecurity policies is both a technical and institutional imperative. Somalia’s progress through the three-pillar framework—specifically the deployment of e-Aqoonsi, HUBIYE, and CDS—reflects meaningful advancement toward a secure digital ecosystem. However, the research indicates that without continued improvements in implementation capacity, digital literacy, and regional regulatory alignment, digital trust will remain limited. As a Somali regulator emphasized, the ultimate success of these initiatives hinges not just on the technology itself, but on whether “citizens believe their data is safe and that the system works in their favor”. Read against the broader digital transformation literature, Somalia’s trajectory illustrates that technical capability alone does not constitute transformation: as institutional and public-sector DT theory suggests ( Mergel et al., 2019; Fountain, 2001), lasting change depends on how new digital infrastructure is enacted through existing bureaucratic capacity, multi-actor coordination, and citizen trust — the very dynamics this study’s three-pillar framework was designed to capture.

The approval of the Cybersecurity Law (2026) and the establishment of SOMCIRT represent a critical milestone in transitioning the nation from a “reactive” to a “proactive” security posture. Together, these reforms provide the legal scaffolding and operational guardrails necessary for securing digital infrastructure and reinforcing public trust. Nevertheless, the long-term impact of these milestones depends on sustained investment in human capacity and institutional coordination.

Based on the analysis and insights gathered from regulators, financial institutions, and civil society, the following policy recommendations are proposed:

▪ Accelerate Regional Harmonization: Collaborate with EAC and continental bodies to align digital ID, data protection, and cybersecurity frameworks, leveraging initiatives such as the Eastern Africa Regional Digital Integration Project (EARDIP) to ensure cross-border interoperability ( Federal Republic of Somalia, 2017), ( East African Community Secretariat, 2022).
▪ Enforce National ID Mandates: Fully implement the Central Bank’s directive to mandate National ID usage across all financial platforms—including mobile money and fintech services—to standardize identity verification and reduce fraud ( Somali National News Agency [SONNA], 2025).
▪ Operationalize Legal Frameworks: Ensure the effective enforcement of the Data Protection Act (2023) and Cybersecurity Law (2026) through the full resourcing of the Data Protection Authority and its integration with SOMCIRT’s incident response mechanisms ( DataGuidance, 2023), ( National Communications Authority [NCA], 2026a), ( National Communications Authority [NCA], 2026b).
▪ Prioritize Rural Digital Literacy: Expand targeted literacy programs in underserved communities to bridge the “awareness gap” identified by stakeholders, ensuring that marginalized populations understand the consent features and protections available to them ( National Communications Authority, 2025).
▪ Achieve Data Sovereignty: Invest in local data centers to transition from foreign-hosted systems to local hosting. This is a prerequisite for strengthening national cybersecurity and mitigating risks associated with external data misuse ( Federal Government of Somalia, 2020).
▪ Strengthen Evidence-Based Policymaking: Establish monitoring and evaluation mechanisms for digital ID usage and public trust indicators. This should include tracking the impact of biometric enrollment on financial inclusion for rural women and other marginalized groups ( National Communications Authority, 2025), ( Republic of Uganda, 2015), ( Federal Government of Somalia, 2023).

Ultimately, Somalia’s experience demonstrates that digital trust is built not only through technological innovation, but through the alignment of legal, institutional, and operational frameworks that place the citizen at the center of the digital transformation.

## Ethics Approval Statement

According to the institutional research guidelines of Somali National University, formal Institutional Review Board (IRB) approval was not required for this minimal-risk qualitative study involving voluntary expert interviews and non-sensitive professional perspectives. The study was conducted in accordance with internationally accepted ethical principles for research involving human participants, including voluntary participation, confidentiality, participant autonomy, and responsible data management.

## Informed Consent Statement

Informed verbal consent was obtained from all participants prior to the interviews and audio recordings. Verbal consent was adopted due to contextual and professional considerations associated with discussions on institutional, policy, and governance-related issues within the Somali context, where written consent could affect participants’ openness and willingness to share professional perspectives. Consent was documented through audio-recorded confirmation and researchers’ field notes. No minors or vulnerable populations were involved in the study.

## Data Availability

The data supporting the findings of this study are not publicly available due to confidentiality and participant privacy considerations. However, anonymized interview data may be made available by the corresponding author upon reasonable request for academic and research purposes. Requests for access to the data may be directed to: Dr. Mohamed Adam Isak, Email: Mohamed.Isak@snu.edu.so Access to the data is subject to ethical considerations and approval by the corresponding author to ensure participant confidentiality is maintained.
