# Supplementary material for: Building Digital Trust in East Africa: The Role of National ID and ICT Policy in Somalia’s Digital Transformation
Source: F1000Res. 2026 Sep 10;15:1029. Originally published 2026 Jun 30. [Version 2] doi: 10.12688/f1000research.182539.2 (PMC13687724; doi:10.12688/f1000research.182539.2)
Supplement: Peer review history

# Reviewer report: Reviewer response for version 2

**DOI:** 10.5256/f1000research.209942.r526586

**Published:** 2026-10-21

**Authors:** Maria de Fátima Pina

**Affiliations:** 1 School of Applied Digital Technologies, ISCTE-University Institute of Lisbon (Ringgold ID: 56061), Sintra, Portugal; 2 Institute of Systems and Robotics, University of Coimbra, Coimbra, Portugal

The article is well structured, with the introduction highlighting the relevance of the research and the objectives defined by three pillars, supported by literature. The methodology section is presented clearly, enabling the study to be replicated. The results are consistent with the objectives, and the research concludes with a summary that does not merely reiterate previous findings, whilst the authors also highlight the implications of the study.

 In the meantime, I would like to offer a few brief comments which I believe might improve the research: firstly, I suggest that the authors remove the paragraphs from the abstract; secondly, only 15 interviews were conducted. Whilst this is acceptable for a qualitative study, it would be interesting to have a more detailed description of the sample’s composition, for example, to know how many consultants and how many digital financial experts were included. Thirdly, the authors acknowledge that they do not have complete data on the reduction in fraud, the impact on financial inclusion, and the actual coverage of rural populations, which means that some conclusions are more forward-looking than empirically substantiated.

 Further, the authors identify the objectives and create a simple conceptual framework within which they present qualitative evidence, concluding with recommendations. They responded clearly to the reviewers, refining the theory and linking it to the three-pillar model. The research is highly creative and relevant, adding value to existing theory. The research may be accepted for indexing, and if the authors deem it relevant, it could be slightly improved by incorporating the comments made.

Is the work clearly and accurately presented and does it cite the current literature?

Yes

If applicable, is the statistical analysis and its interpretation appropriate?

Yes

Are all the source data underlying the results available to ensure full reproducibility?

Yes

Is the study design appropriate and is the work technically sound?

Yes

Are the conclusions drawn adequately supported by the results?

Yes

Are sufficient details of methods and analysis provided to allow replication by others?

Yes

Reviewer Expertise:

STEM areas

I confirm that I have read this submission and believe that I have an appropriate level of expertise to confirm that it is of an acceptable scientific standard.

# Reviewer report: Reviewer response for version 2

**DOI:** 10.5256/f1000research.209942.r520109

**Published:** 2026-09-22

**Authors:** Abeer F. Alkhwaldi

**Affiliations:** 1 Dakota State University, Madison, South Dakota, USA

The authors addressed all the required comments

Is the work clearly and accurately presented and does it cite the current literature?

Yes

If applicable, is the statistical analysis and its interpretation appropriate?

Yes

Are all the source data underlying the results available to ensure full reproducibility?

Yes

Is the study design appropriate and is the work technically sound?

Yes

Are the conclusions drawn adequately supported by the results?

Yes

Are sufficient details of methods and analysis provided to allow replication by others?

Yes

Reviewer Expertise:

Digital Transformation

I confirm that I have read this submission and believe that I have an appropriate level of expertise to confirm that it is of an acceptable scientific standard.

# Reviewer report: Reviewer response for version 1

**DOI:** 10.5256/f1000research.201492.r515333

**Published:** 2026-09-02

**Authors:** Abeer F. Alkhwaldi

**Affiliations:** 1 Dakota State University, Madison, South Dakota, USA

This manuscript provides a timely and detailed empirical investigation into Somalia’s evolving digital ecosystem. Using a qualitative multi-method approach combining 15 expert interviews with legal and strategic policy analyses the authors explore how digital identity platforms (e-Aqoonsi, HUBIYE, CDS), regulatory frameworks (Data Protection Act 2023), and cybersecurity infrastructure (Cybersecurity Law 2026, SOMCIRT) intersect to foster digital trust and financial inclusion. The conceptual "Three Pillars" framework effectively grounds the study, and the comparative assessment with East African Community (EAC) peers highlights key operational realities, such as data sovereignty challenges and rural digital literacy gaps.

**Key Strengths**

- **Contextual Significance:** Offers valuable insights into digital transformation in fragile state settings, an area often underrepresented in scholarly literature.
- **Methodological Triangulation:** Successfully balances primary stakeholder perspectives with a rigorous evaluation of key legislative texts.
- **Policy Relevance:** The strategic recommendations directly address core institutional barriers, such as local data hosting infrastructure and regional regulatory harmonization.

**Areas for Improvement & Literature Integration:**

 While the paper is well-structured, expanding its grounding in broader academic literature would strengthen its analytical contribution:

- **Expand Conceptual Grounding on Digital Transformation:** The manuscript evaluates digital transformation primarily through a policy and legal lens. Incorporating broader scholarly literature on digital transformation dynamics specifically how socio-technical regimes, public-private actor networks, and institutional capabilities shape tech adoption in emerging economies would deepen the theoretical foundation.

Papers that are recommended to be cited:

- https://doi.org/10.1108/IJLMA-11-2023-0249
- doi: 10.1109/ICETSIS61505.2024.10459639
- doi: 10.1109/ICBATS57792.2023.10111266

 

- **Cite Core Digital Transformation Literature:** Consider engaging with established foundational and contemporary studies on digital transformation, enterprise digital maturity, and technology-driven public sector modernization. Contextualizing Somalia’s trajectory within wider theoretical debates on digital government evolution and ICT-led socio-economic change will elevate the paper's academic contribution beyond its current policy focus.
- **Refine Empirical Impact:** If possible, include preliminary metrics or early usage estimates regarding e-Aqoonsi enrollment to complement the qualitative stakeholder insights.

**Recommendation**

**Minor Revision.** The paper offers strong empirical value and clear strategic roadmaps. Engaging more directly with broader academic literature on digital transformation will make this contribution even more impactful for researchers and policymakers alike.

Is the work clearly and accurately presented and does it cite the current literature?

Yes

If applicable, is the statistical analysis and its interpretation appropriate?

Yes

Are all the source data underlying the results available to ensure full reproducibility?

Yes

Is the study design appropriate and is the work technically sound?

Yes

Are the conclusions drawn adequately supported by the results?

Yes

Are sufficient details of methods and analysis provided to allow replication by others?

Yes

Reviewer Expertise:

Digital Transformation

I confirm that I have read this submission and believe that I have an appropriate level of expertise to confirm that it is of an acceptable scientific standard, however I have significant reservations, as outlined above.

## References

[1] : Digital transformation in financial industry: antecedents of fintech adoption, financial literacy and quality of life. *International Journal of Law and Management* .2025;67(6) : 10.1108/IJLMA-11-2023-0249697-723 10.1108/IJLMA-11-2023-0249

[2] A. Abu-AlSondos, M. Shehadeh, M. Ajouz, A. F. Alkhwaldi, M. Abdeldayem and S. H. Aldulaimi, "The Role of Digital Transformation in Business: Opportunities Challenges and Future Directions," 2024 ASU International Conference in Emerging Technologies for Sustainability and Intelligent Systems (ICETSIS), Manama, Bahrain, 2024, pp. 361-365, doi: 10.1109/ICETSIS61505.2024.10459639.

[3] M. Shehadeh, D. Almajali, I. A. Abu-AlSondos, A. F. Alkhwaldi and A. S. Al-Gasaymeh, "Digital Transformation and its Impact on Operational Efficiency and Competitive Advantage in Islamic Banks," 2023 International Conference on Business Analytics for Technology and Security (ICBATS), Dubai, United Arab Emirates, 2023, pp. 1-6, doi: 10.1109/ICBATS57792.2023.10111266.

# Response

**Published:** 2026-09-03

**Authors:** Mohamed Adam Isak

**Affiliations:** Computer Science, Somali National University, Mogadishu, Benadir, Somalia

Dear Reviewer,

 Thanks for taking the time to read the manuscript and for your insightful remarks. We are delighted that the Three Pillars framework and the EAC comparative assessment were well received and we have amended the manuscript (Version 2) in response to your recommendations.

**Broadening the theoretical underpinning of digital transformation.**

 We agree that the initial contribution focused primarily on digital transformation from the point of view of policy and law. We have added a new Literature Review section, entitled “Theoretical Basis of Digital Transformation”, that situates Somalia’s reforms in the broader socio-technical and institutional literature you identified, including all three of your recommended citations: Alkhwaldi (2025) on antecedents of fintech adoption and digital trust, Abu-AlSondos et al. (2024) on opportunities of digital transformation and organisational challenges, and Shehadeh et al. (2023) on the impact of digital transformation on operational efficiency in Islamic banks. The last two studies were especially pertinent to our consideration of KYC and account opening processes considering the mostly Sharia compliant financial sector in Somalia.

**More generally mentioning core literature on digital transformation.**

 The new subchapter also addresses prior fundamental and present work on digital transformation dynamics: Vial (2019) on digital transformation as value-creation change Mergel, Edelmann and Haug (2019) on public-sector digital transformation Fountain (2001) on technology enactment theory Meijer and Bolívar (2016) on multi-actor digital governance networks Heeks (2020) on the ICT4D 3.0 “digital-for-development” paradigm. In the Conclusion, we have also included a final paragraph that directly connects these theoretical issues to our three-pillar framework, situating the paper’s empirical findings in broader conversations about the growth of digital government and institutional competence.

**Refining empirical impact with early usage estimates**

 We add a paragraph with preliminary enrolment data for e-Aqoonsi in the public domain (over one million registrations as at November 2025, independent reporting) and more recent budget and target figures announced in January 2026. These figures are not derived from NIRA’s own disaggregated internal statistics, but from public statements, and hence we have expressly highlighted them as indicative rather than precise, but they do verify our qualitative conclusion of persistent rural under-registration.

 We believe that these improvements further enrich the theoretical contribution of the manuscript, while maintaining its original empirical and policy focus, and thank you again for assisting us to sharpen the paper’s engagement with the digital transformation literature.

 Yours sincerely,

 Mohamed Adam Isak (on behalf of the authors)
